# Physical unclonable function using photonic spin Hall effect

**DOI:** 10.1038/s41598-024-65176-0

**Published:** 2024-06-22

**Authors:** Divyanshu Divyanshu, Amit Kumar Goyal, Yehia Massoud

**Affiliations:** Innovative Technologies Laboratories (ITL), King Abdullah University of Science and Technology (KAUST), 23955 Thuwal, Saudi Arabia

**Keywords:** Photonic spin Hall effect (PSHE), Photonic crystals (PhC), Physically unclonable functions (PUFs), Photonic crystals, Nanophotonics and plasmonics, Electronic and spintronic devices

## Abstract

This study presents a novel method leveraging surface wave-assisted photonic spin Hall effect (PSHE) to construct physical unclonable functions (PUFs). PUFs exploit inherent physical variations to generate unique Challenge–Response pairs, which are critical for hardware security and arise from manufacturing discrepancies, device characteristics, or timing deviations. We explore PSHE generation-based PUF design, expanding existing design possibilities. With recent applications in precise sensing and computing, PSHE offers promising performance metrics for our proposed PUFs, including an inter-Hamming distance of 47.50% , an average proportion of unique responses of 62.5% , and a Pearson correlation coefficient of − 0.198. The PUF token demonstrates robustness to simulated noise. Additionally, we evaluate security using a machine learning-based attack model, employing a multi-layer perceptron (MLP) regression model with a randomized search method. The average accuracy of successful attack prediction is 9.70% for the selected dataset. Our novel PUF token exhibits high non-linearity due to the PSHE effect, resilience to MLP-based attacks, and sensitivity to process variation.

## Introduction

Hardware security encompasses practices aimed at safeguarding physical components within various systems from unauthorized access and tampering. It includes physical security measures, tamper resistance techniques, secure boot processes, trusted platform modules (TPM), hardware-based encryption, and firmware protection to defend against diverse threats^1^. Physical unclonable functions (PUFs) enhance device authenticity and resistance to cloning by generating unique identifiers based on inherent physical variations^2^. Cryptography, integral to hardware security, involves dedicated hardware components for encryption and decryption processes, secure key storage, and protection against side-channel attacks. Traditional hardware for such applications has relied on conventional CMOS technology. However, novel technologies are being explored to enhance PUF performance, leveraging unique characteristics such as those based on spintronics and photonics. In spintronics, PUFs exploit the unique magnetic properties of electrons, utilizing spin states to generate distinct and non-reproducible identifiers^3,4^. Photonics-based PUFs, on the other hand, utilize light characteristics such as optical phase or intensity fluctuations to create unique fingerprints for each device. Optical PUFs were first proposed by Pappu et al.^5^, the authors show that the mesoscopic physics of coherent transport through a disordered medium can be used for constructing PUFs. Grubel et al.^6^ experimentally demonstrated Photonic PUFs using the ultrafast non-linear optical interactions in a chaotic silicon micro-cavity. In the year 2022, Photonic PUFs using active quasi-crystal interferometers with integrated micro-heaters were proposed by Tarik et al.^7^. Several other designs and physical characteristics have been used in designing optical PUFs in recent years^8–12^. Both spintronics and photonics PUFs offer a high level of security due to intrinsic physical variations that are challenging to replicate. By harnessing spin/light principles, these PUF implementations provide robust and tamper-resistant solutions, making them valuable components in safeguarding sensitive information and ensuring device authenticity across various applications.

Analogous to utilizing the spin hall effect in electrons, researchers are working towards enhancing the transverse displacement of the photonic spin hall effect (PSHE). The PSHE effect arises from the spin–orbit interaction of light, orbital angular momentum, and geometric phases^13^. It manifests as a spin-dependent transverse shift of photons concerning the geometric optical trajectory when the beam traverses an optical interface or inhomogeneous medium^14,15^. The first experimental validation of PSHE at an air-glass interface was demonstrated by Hosten et al.^16^. Subsequently, various advancements and techniques have been proposed in the context of PSHE and its applications^17^. In our recent work, we proposed a 1D-photonic crystal (1D-PhC) assisted optical tamm state (OTS) excitation for PSHE enhancement and reported a 10.73 $\times \,\lambda$ (or 6.78 $\upmu$m) PSHE-based transverse displacement at a constant incidence angle^18^. Thus, analogous to the spin hall effect of electrons in designing conventional spintronics device-based PUFs, the PSHE can also be considered a viable alternative for unconventional hardware security primitives for logic locking^19^, hardware watermarks^20^, and encryption engines^21^.

**Figure 1:**
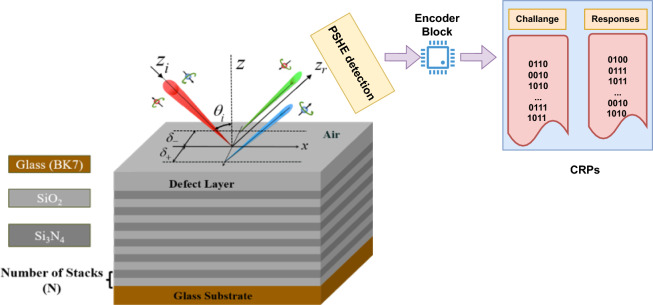
Block diagram representation of the proposed PUF working method.

Therefore, the present research provides a novel PSHE-based approach to creating optical PUFs. This study generates challenge–response pairs (CRPs) based on the PSHE effect for PUF token design. Figure 1 illustrates the proposed setup for detecting the PSHE effect and generating CRPs. The chosen 1D-PhC structure, composed of alternating SiO$_{{2}}$ and Si$_{{3}}$N$_{{4}}$ layers with a top defect layer, facilitates PSHE shift detection (typically a few micrometers). The PSHE detection usually involves measuring the spatial separation of optical spins induced by spin–orbit coupling in the system. The measurement can be done using various techniques such as near-field scanning microscopy^22^, interferometry^23^, direct imaging of the spin-dependent intensity distribution^24^, or weak measurements^25^ for small PSHE shifts (few nanometers). The encoder block will convert the measured value and generate CRP pairs using a suitable encoding method for performance analysis.

The PUF exhibits strong non-linearity in the CRP dataset and resilience to introduced noise and process variation in defect layer thickness, indicating robustness to noise and limiting cloning attack success rates. However, it displays weaker performance in achieving uniform distribution within the CRP dataset, attributed to a small full-width-half-maximum (FWHM) at $\frac{\mid r_{TE}\mid }{\mid r_{TM}\mid }$ $\approx$ 0.5 of approximately $0.003^\circ$ for the PSHE effect, posing a challenge during dataset encoding. Additionally, the PUF’s security is evaluated using an ML-based model, with over 1000 iterations of ’Random-Search’. This results in a correct prediction rate of only 9.70% for the CRP dataset, indicating resilience against ML-based attack models aimed at deciphering the PUF’s token properties.

The rest of the paper is organized as follows: Section “Background” gives relevant information for this work. Section “Design of PSHE-based PUFs using 1D-PhC” outlines the methodology and showcases the results. Finally, The “Conclusion” section offers concluding remarks on the study.

## Background

The 1D-PhC, a photonic crystal exhibiting periodic refractive index variation in a single dimension, comprises alternating high and low refractive index regions^26^. Introducing a defective layer in the regular 1D-PhC (Bragg reflector) generally creates sharp and narrow polarization-dependent reflection valleys for *s* or *p* waves at a constant incident angle^27,28^. For generating enhanced PSHE-based transverse shift in the beam centroid, a larger value of Fresnel reflection ratio is required^29^.

### Theoretical analysis of PSHE using OTS excitation

**Figure 2:**
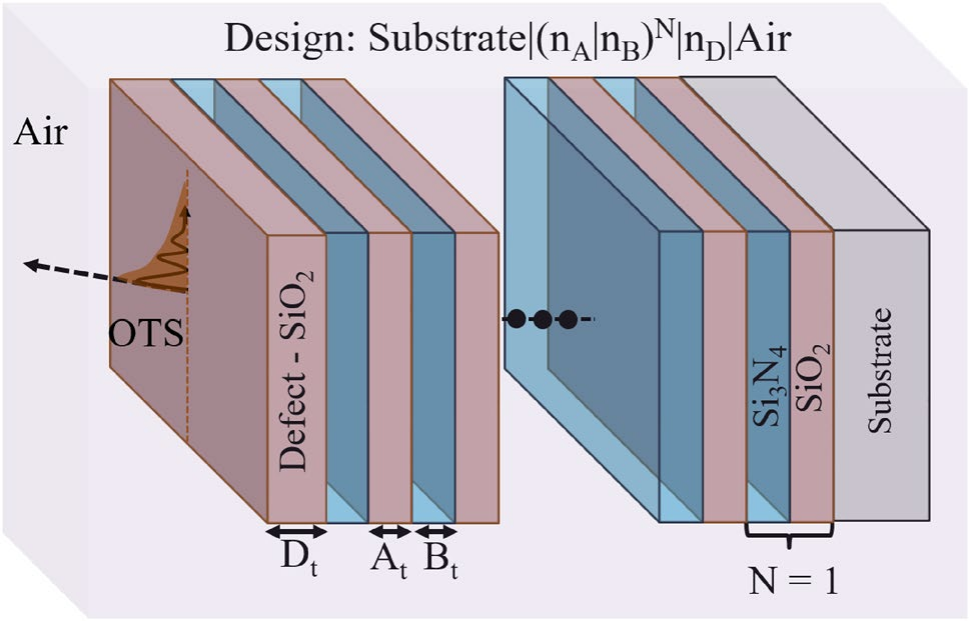
Schematics of proposed dielectric 1D-PhC structure to excite OTS.

Figure 2 provides the schematics of the proposed 1D-PhC structure, with the configuration: [Substrate(Glass)$\mid$($A_{\text{SiO}_{{2}}}$ $B_{\text{Si}_{{3}}\text{N}_{{4}}}$)$^N$ $\mid$ Defect (D)$\mid$Air]. Here, thickness of layer ‘A’ ($A_{t}$) is 128 nm, and that of layer ‘B’ ($B_{t}$) is 85 nm, corresponding refractive indices with their optical extinction coefficient are: $n_{A}$ = 1.46, $n_{B}$ = 2.2, $k_{A}$ = 0, and $k_{B}\,=\,-0.0002$. The periodic repetition value (N) is set at 7. The device demonstrates a critical defect layer thickness of about 150 nm below that, no OTS is excited as the excited mode approaches the air light line for operating wavelength; more discussion on the generation method and design analysis is provided in our previous work^18^. The defect layer is selected as $\text{SiO}_{2}$. Further, for defect layer thickness ($D_{t}$) $\in$ (150 nm, 200 nm), $\theta _{i}$ $\ge$ $\theta _{critical}$ (Critical angle $\approx$ 41$^\circ$) OTS propagation is sustained. The range where the CRP dataset can be generated is under the designer’s control and can be reconfigured by changing the underlying parameters. This can be considered Intellectual Property (IP) for the designer, which may add extra security against Reverse Engineering (RE) or other modeling attacks. In order to establish the dispersion relation for the OTS and to solve the eigenvalue problem, the Floquet theorem is used^30^:

1 $$\begin{aligned} K(\beta , \omega )=\frac{1}{\Lambda } \cos ^{-1}\left( \frac{1}{2}\left( M_1+M_4\right) \right) \end{aligned}$$

Here, $M_{n}$ represents the eigenvalue matrix elements, whereas the Bloch wave vector is represented by ‘K’. The structure periodicity is represented by $\Lambda$, which is repeated ‘N’ times. The real values of the Bloch wave vector are attributed to the propagating OTS, and imaginary values give information on evanescent OTS. The transfer matrix is formulated to calculate the reflected and transmitted wave amplitude^31^.

When a monochromatic Gaussian beam of wavelength $\lambda$ having beam waist of $W_{0}$ is incident on a multilayer structure like 1D-PhC, the corresponding angular spectrum can be described by Eq. (2). This incident beam is divided into two circularly polarized components due to PSHE.

2 $$\begin{aligned} \tilde{\textbf{E}}_{i \pm }=\left( \textbf{e}_{i x} \pm i\textbf{e}_{i y}\right) \frac{W_0}{\sqrt{2 \pi }} \exp \left[ -\frac{W_0^2\left( k_{i x}^2+k_{i y}^2\right) }{4}\right] , \end{aligned}$$

here, $k_{ix}$ and $k_{iy}$are the wave-vector components in the $x_{i}$ and $y_{i}$ direction, and $+/-$ represents the corresponding left and right circular polarization components. The PSHE-based shift ($\delta$) with regards to geometric optic prediction is given by Eq. (3)^32^.

3 $$\begin{aligned} \delta _{\pm }^{V, H}=\frac{\iint \tilde{\textbf{E}}^* i \partial _{k_{r y}} \tilde{\textbf{E}} d k_{r x} d k_{r y}}{\iint \tilde{\textbf{E}}^* \tilde{\textbf{E}} d k_{r x} d k_{r y}} . \end{aligned}$$

Equation (3) is simplified, and the detailed theoretical description is provided in^33^:

4 $$\begin{aligned} \delta _{\pm }^{V}=\mp \frac{k W_0^2 \textrm{R} e\left( 1+r_p / r_s\right) \cot \theta _i}{k^2 W_0^2+\left| \frac{\partial l n r_s}{\partial \theta _i}\right| ^2+\left| \left( 1+r_p / r_s\right) \cot \theta _i\right| ^2}, \end{aligned}$$

Here, $r_{s}$ and $r_{p}$ is the Fresnel reflection coefficient for the *s* and *p*, polarized light, respectively, and $\theta _{i}$ is the incident angle. $\left| \frac{\partial l n r_s}{\partial \theta _i}\right| ^2$ $\approx$ 0, allows Eq. (4) to be simplified further, solving some mathematical inequalities results in^25,34^:

5 $$\begin{aligned} \delta _{\pm }^{V}=\mp \left( 1+{\text{Re}}\left[ r_p\right] / {\text{Re}}\left[ r_s\right] \right) \cot \theta _{i} / k \end{aligned}$$

The structure shows a extremely small FWHM of around 0.003$^\circ$ at $\frac{\mid r_{TE}\mid }{\mid r_{TM}\mid }$ $\approx$ 0.5. This shows a very narrow region for generating a sufficiently large PSHE shift. The greater reflection sensitivity towards narrower angle dependency is utilized to produce device-specific PSHE shifts and generate unique CRP sets, a key requirement in PUF design.

## Design of PSHE-based PUFs using 1D-PhC

**Figure 3:**
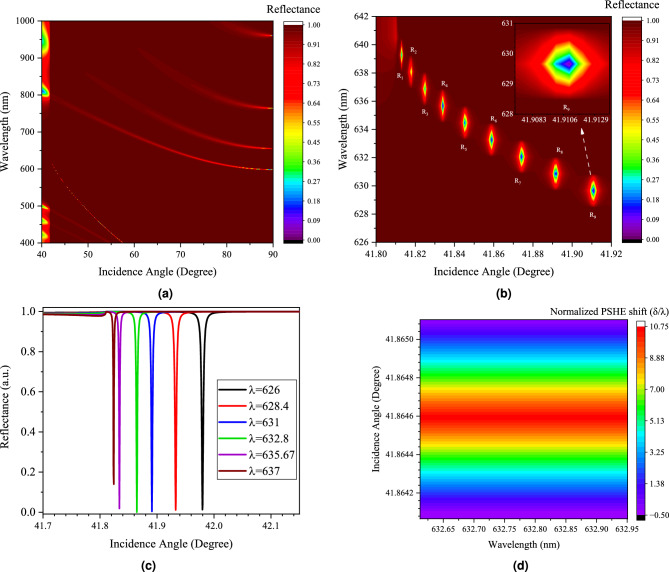
(**a**,**b**) The angular dispersion characteristics of proposed structure at $D_{t}$ = 155.8 nm, (**b**) Enlarged dispersion characteristics indicating response data, (**c**) Corresponding reflectance spectrum at six different operating wavelengths, and (**d**) Normalized PSHE shift.

The proposed optimized structure exhibits the OTS excitation at $\theta _{i}$ = 41.86$^\circ$ for TE polarization and is considered as an initial parameter, which is shown in Fig. 1. Due to the PSHE effect, the beam splits into corresponding optical spins, when the incident light falls upon the device. The sensor block senses the PSHE shift and can send the stored data to the encoder block to generate the response. The structure is optimized to provide a PSHE shift of 6.78 $\upmu$m, with defect layer thickness ($D_{t}$) of around 155 nm. Figure 3 shows the analytical modeling of PSHE generation on the selected PUF instance. Figure 3a shows wavelength-dependent angular dispersion analysis of the proposed structure with corresponding reflectance value for TE polarized light. This dispersion characteristic is device-dependent, ensuring the CRP dataset’s uniqueness. In Fig. 3b, response data ($R_{1}$ to $R_{9}$ is shown) are extracted for a particular challenge bit stream at different instances of $\theta _{i}$. These exceptional points at which response data is collected are based on the PSHE effect and provide a particular set of reflection values, with few responses showing the maximum reflectance ratio ($R_{TE}$ $\approx$ 0 and $R_{TM}$ $\approx$ 1). This behavior can further be utilized in cryptographic applications requiring a definite PSHE shift. A total of 15 response datasets are extracted for proof of concept demonstration. A large number of Response files can be collected along the OTS propagation line. Figure 3c gives a different visualization of the reflectance response for slight variations of wavelength-incident angle pair. This indicates the robustness of OTS excitation. In Fig. 3d, the analytical modeling provides the normalized PSHE shift displacement value up to 10.73 times incident wavelength. This provides sufficient displacement resolution for the practical encoding of PSHE shift. Moreover, the excited OTS and PSHE-based transverse displacement is highly susceptible to variations in defect layer thicknesses.

**Figure 4:**
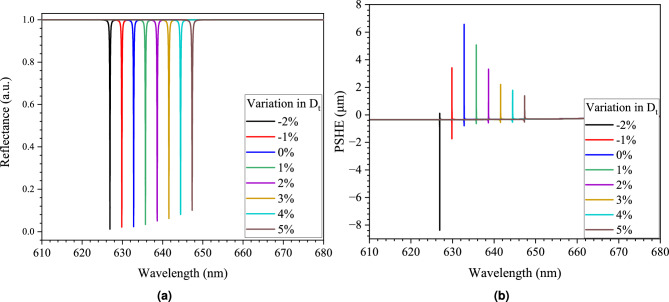
Wavelength dependent impact of defect layer thickness variations (− 2% to 5%) on (**a**) OTS generation, and (**b**) PSHE generation.

Figure 4a shows the OTS excitation for different values of process variation in $D_{t}$ at a constant incident angle of 41.86$^\circ$. The figure shows that despite slight variation in $D_{t}$, the OTS is excited at different operating wavelengths, which results in corresponding PSHE generation. This high sensitivity to the structure’s optical parameters is helpful for the possibility of high-performance PUFs using photonic crystals. CRP sensitivity to process variation is an essential characteristic of PUFs as it thwarts cloning-based attacks, where the attacker gains insight into the PUF token using sophisticated engineering techniques such as scanning electron microscopy or Transmission electron microscopy imaging. This high sensitivity can also provide a measure against tampering-based attacks. Some tampering-based attacks are classified as destructive methods^35^; due to their destructive nature, they are generally error-prone, and thus, physical tamper-based attacks may not be very successful, as they will alter the CRP dataset significantly. Figure 4b shows the PSHE shift displacement variation at specific wavelengths for a fixed $\theta _{i}$ of 41.86$^\circ$. The result demonstrates high non-linearity in PSHE generation with variation in $D_{t}$; this property allows for better resilience against ML-based attack models. Non-linear relationship is a critical requirement in PUFs as it increases the computational cost of ML-based attack models to decipher and predict PUF responses.

In the context of PUFs, there are several well-defined performance metrics. Depending on the particular application, the PUF metrics can give an idea of its usefulness. The device performance metrics are evaluated using the CRP dataset, and the advantages and current limitations of the work are pointed out based on it. A CRP set is needed to assess the PUF’s performance metrics and attack resilience ability. The following subsection describes the adopted methodology in detail and provides relevant information.

### PUF performance metrics

CRP data is generated using an adaptive encoding method. The encoding method considers the reflectance value for the specific challenge (here, wavelength of the incident light is coded in binary format). The encoded challenge set ($C_{X}$) is thus dependent on two parameters of the incident light, wavelength, and incident angle, i.e., $C_{X} = f(\lambda , \theta _{i})$. Other parameters like intensity and beam waist may also be selected. The response set ($R_{Y}$) contains $N_{R}$ = 15 Response data set ($R_{1}$ to $R_{15}$), each containing, $m_{R}$ = 8 response points of $n_{b}$ = 4 bits length sampled at different instances of $\theta _{i}$, according to Fig. 3b. A large dataset can be generated along the propagation line, and a small set is taken here to demonstrate the proof of concept and the analytical behavior.

The following PUF metrics are evaluated for the proposed structure.

#### Entropy

Entropy measures the randomness or unpredictability of the responses. Higher entropy indicates better unpredictability. As the response data is in binary, Eq. (6) is used for calculation of Shannon Entropy of the system (*H(X)*):

6 $$\begin{aligned} H(X)=-p_0 \log _2\left( p_0\right) -p_1 \log _2\left( p_1\right) \end{aligned}$$

Here, $p_{0}$ and $p_{1}$ indicate the probability of occurrence of bits ‘0’ and ‘1’ in the entire response data set.

#### Linearity

Non-linearity is often desired to enhance security by making it difficult to predict the response to a new challenge based on the responses to known challenges. Here, we first compute the Pearson relation coefficient between $C_{X}$ and $R_{Y}$ based on Eq. (7). The value of the Pearson relation Coefficient is always $\mid$r$\mid$ $\le 1$, where value r = 0 indicates no linear relationship and $\mid$r$\mid$ = 1 indicates a linear relationship.

7 $$\begin{aligned} r=\frac{\sum _{i=1}^{N_R}\left( C_{X_i}-\bar{C_{X}}\right) \left( R_{Y_i}-\bar{R_{Y}}\right) }{\sqrt{\sum _{i=1}^{N_R}\left( C_{X_i}-\bar{C_{X}}\right) ^2 \sum _{i=1}^{N_R}\left( R_{Y_i}-\bar{R_{Y}}\right) ^2}} \end{aligned}$$

Here, $C_{X_i}$ and $R_{Y_i}$ are individual observations of $C_{X}$ and $R_{Y}$, $\bar{C_{X}}$ and $\bar{R_{Y}}$ is the means of $C_{X}$ and $R_{Y}$, respectively.

#### Inter-hamming distance

In the context of PUFs, inter-hamming distance refers to the average Hamming distance calculated across pairs of responses. The ideal value is 50%. It is calculated by using Eq. (8) as shown:

8 $$\begin{aligned} H.D._{\text{inter} }=\frac{1}{N_R \cdot (N_R-1)} \sum _{i=1}^{N_R} \sum _{j \ne i} {\text{H.D.}}\left( R_i, R_j\right) \end{aligned}$$

Here, *H.D.(*$R_{i}$, $R_{j}$*)*, represents the hamming distance between $R_{i}$ and $R_{j}$.

### Results and discussion

**Figure 5:**
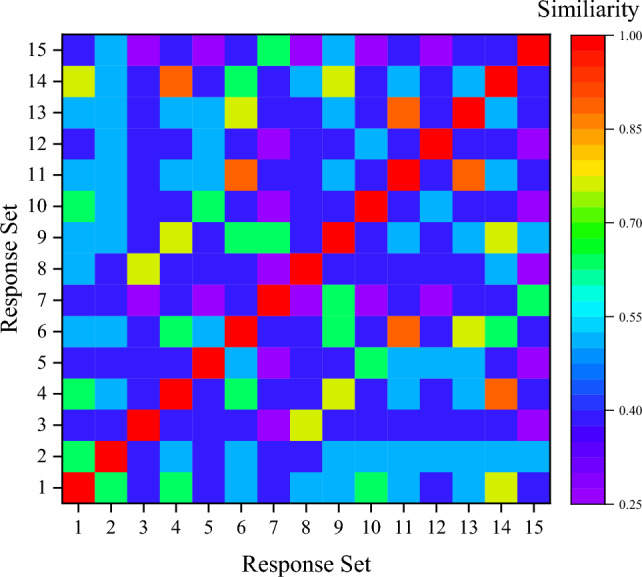
Response similarity matrix representation.

First, we provide a detailed analysis of the dataset taken for $R_{Y}$ after the encoding method. Figure 5 shows the response similarity matrix obtained for $R_{Y}$. The visualization provides the randomness and complexity associated with the Response set. An average Response similarity of 11.22% is obtained for $R_{Y}$. This indicates a greater dissimilarity within $R_{Y}$. The average proportion of bit ‘1’ obtained in the dataset is 37.01%. Ideally, this value should be 50%. The average proportion of unique responses is 62.5%. The average Shannon entropy (*H(X)*) of $R_{Y}$ using Eq. (6) is 2.05. The average Pearson relation coefficient (r) obtained based on Eq. (7) is − 0.198, indicating a highly non-linear relationship within $R_{Y}$.

**Figure 6:**
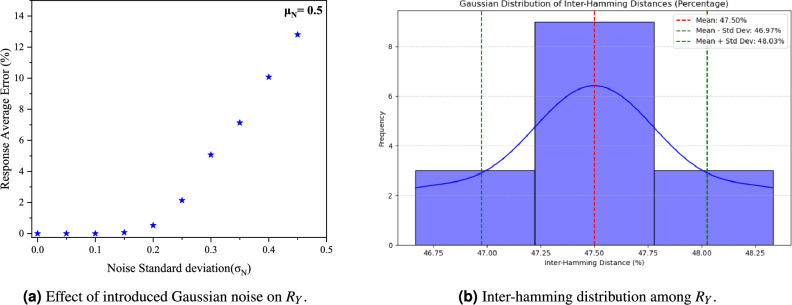
Simulation results related to performance metrics for the proposed PUF token.

In Fig. 6a, a Gaussian noise with a mean value of 0.5 (due to binary data format) is added to record the response average error for varying standard deviation (SD) in the noise; the result indicates about 6% error in value till 0.3 SD in noise value. This shows the response dataset’s robustness regarding tolerance to introduced error. Figure 6b shows the Inter-Hamming distance between response sets calculated using Eq. (8), a mean value of 47.50% is obtained. It is noteworthy to mention that the PVT tests, which refers to process, voltage, and temperature testing is essential in conventional electronic circuit design, may not be suitable for standalone PhC-PUFs using PSHE as the proposed devices work by incidenting light beam not using Voltage sources. Moreover, the thermos-optics coefficient of the considered materials is very less (in the range of 10$^{-4}$ K$^{-1}$). Thus, the generated CRPs are not a strong function of temperature. Due to the unique nature of the proposed PUF using PSHE, direct comparison with other PUFs is challenging due to variations in architectures, device characteristics, entropy sources, performance metrics, and a range of CRP data sets taken with different generation/sampling techniques. Table 1 provides a qualitative comparison with other recently proposed Optical PUFs.

**Table 1:** Qualitative comparison table with recently proposed Optical PUFs.

PUF token	Physical basis	Performance evaluated	Limitations	Year
Disordered photonic structure^9^	Scattered distribution of electromagnetic field	Uniqueness, and tamper resistance	ML-attack evaluation, analytical/theoretical	2016
Chaotic silicon micro-cavity^6^	Ray chaotic behavior	Reproducibility, uniqueness, unclonability	ML-attack evaluation	2017
Multi-mode optical waveguide^10^	Physical scrambling mechanisms	Robustness, uniqueness, unclonability	ML-attack evaluation, bulk Optical Component	2018
Silicon disk resonators^36^	Plasmonics enhanced nanoparticles	Randomness, uniqueness, reproducibility	ML-attack evaluation, analytical/theoretical	2019
Quasicrystal interferometer^7^	Use of integrated micro-heaters, grating couplers	Uniqueness, and reconfiguration	ML-attack evaluation	2022
PhC (This work)	Photonic spin Hall effect	Entropy, linearity, uniqueness, ML-attack evaluation	Analytical/theoretical	2024

### Performance against ML-based attacks

PUFs offer the potential for enhanced hardware security. In many cases, attackers can physically access the PUF token, which opens up the possibility for ML-based modeling attacks. These attacks involve the attacker attempting to extrapolate the behavior of the PUF token to predict its future behavior, or they can deduce the IP of the PUF token under consideration based on the CRP data set along with RE methods. If successful, the attacker could create a predictive model indistinguishable from the original PUF token, compromising its security.

**Figure 7:**
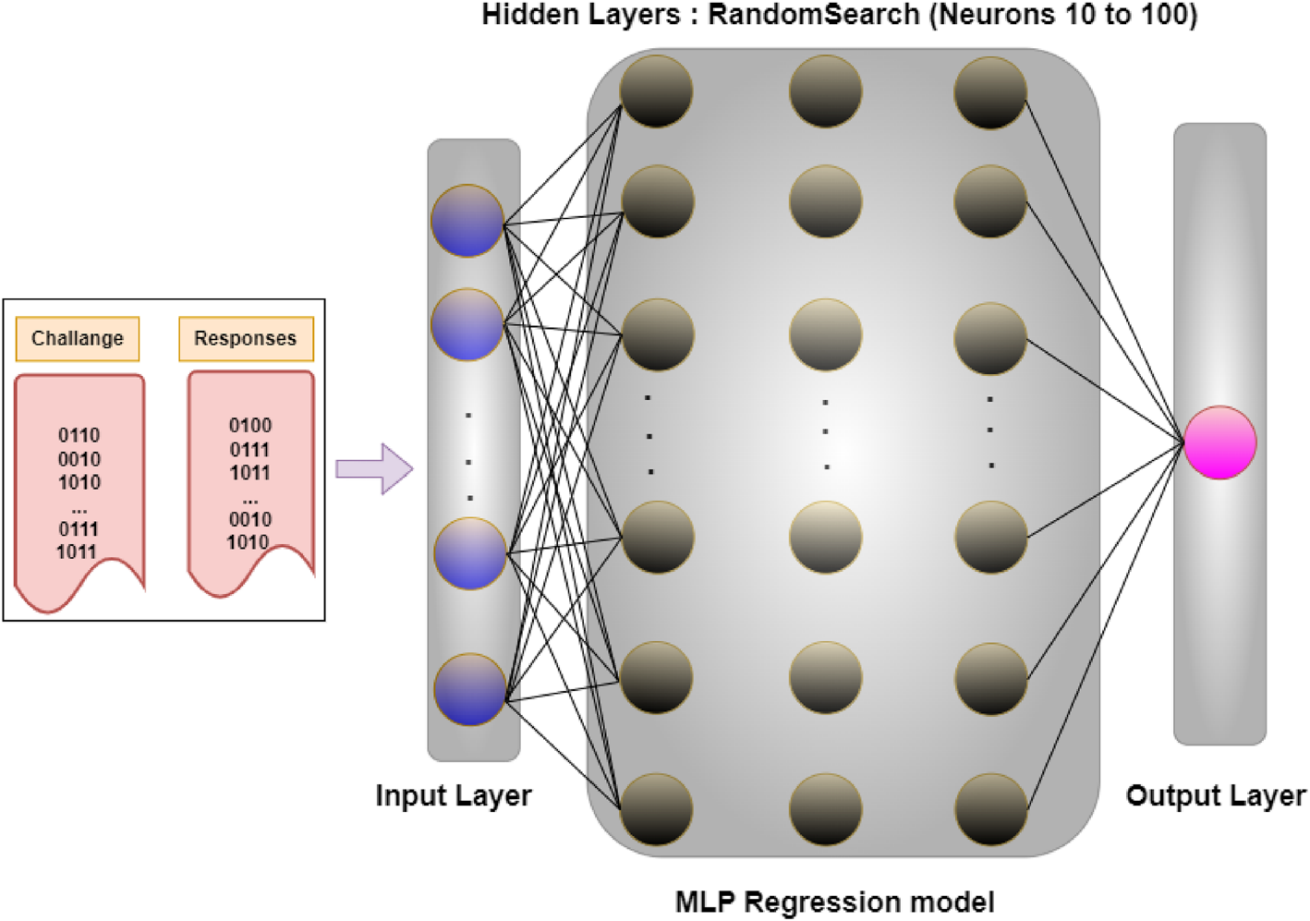
MLP regression model neural network architecture representation.

**Figure 8:**
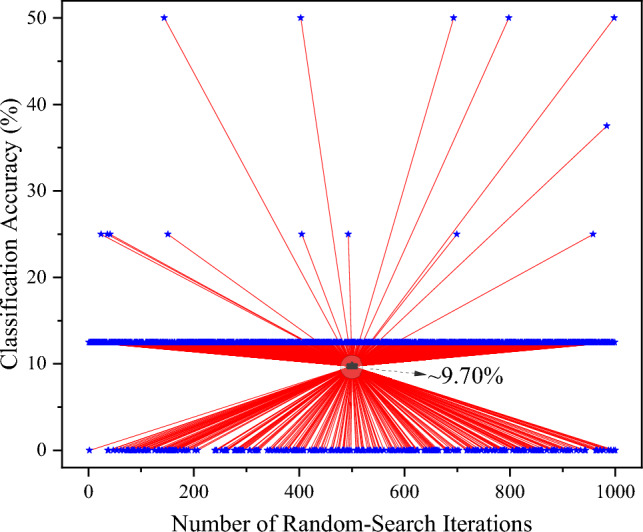
Classification accuracy results for 1000 iterations of ‘random-search method’ with three hidden layers and varying architecture hyper-parameters.

To evaluate the performance of our proposed PUF model for the selected dataset, the challenges are scaled using ‘StandardScaler’ method. Parameter distributions are defined for a randomized search method to optimize hyper-parameters for a multi-layer perceptron (MLP) regressor model. The network architecture is shown in Fig. 7. The number of hidden layers = 3, and within each hidden layer, the number of neurons varies from 10 to 100 during the random-search method. A total number of 1000 iterations is performed for the random-search method to obtain the overall correct prediction of responses as shown in Fig. 8.

The randomized search with cross-validation is used to determine the best MLP regressor model, considering parameters such as hidden layer sizes, regularization parameters, and initial learning rate. A sigmoidal activation function is used for individual neurons. From Fig. 8, an average accuracy of correct prediction of 9.70% is obtained. The maximum accuracy of 50% is achieved for 0.005% of simulations during the randomized search. The best parameter is obtained for the following configuration during the search (Regularization parameter = 0.01, Learning rate = 0.3). This data indicates the difficulty of the MLP regression model in accurately predicting the selected CRP dataset. This is due to the high non-linearity and response complexity in $R_{Y}$. Table 2 provides qualitative information about some ML-based modeling attack methods on PUF structures.

**Table 2:** Qualitative comparison for ML-based attack model on some PUF structure.

ML-method	Targeted PUF	Features leveraged	Potential countermeasure	Attack difficulty	Year
Logistic regression^37^	Electrical strong PUF	CRP dataset	CRP obfuscation	Low	2010
Adaptive RProp-ML^38^	XOR-Arbiter PUF, lightweight PUF	CRPs; time and power side channel analysis	Dynamic/differential CMOS logic, prevent repeated measurements	Medium	2014
CMA-ES ML^39^	Strong PUF-based RFID tag	CRPs; reliability or bias side channels	CRP lockdown, prevent repeated measurements	Low	2015
Lattice basis and BKZ reduction^40^	XOR-arbiter PUF	CRPs; photonic side channel analysis	Interconnect meshes and optical interaction, prevent repeated measurements, 3D integration	High	2015
MLP regression (This work)	PSHE-based PhC PUF	CRP dataset	CRP obfuscation and CRP lockdown	High	2024

The findings indicate strong performance, particularly in scenarios with high non-linearity, increased response complexity, elevated entropy, and sensitivity to process variations. The intricate generation mechanism of the PSHE and the structure’s susceptibility to process variation suggest potential resistance against cloning and tampering attacks. However, optical PUFs are typically non-silicon PUFs, limiting their electronic compatibility. Furthermore, the proposed device might exhibit sensitivity to environmental factors like humidity, potentially affecting its reliability. Additionally, degradation over time could impact its performance. As this work is analytical, the evaluation of such parameters is limited in this work. Based on analytical simulation, the encoding method demonstrates weaker performance in terms of even distribution of bits and inter-hamming distances in $R_{Y}$. Improvement in these parameters will allow for more secure PUF structures in the future. The proposed structure has a more straightforward fabrication methodology, like spin/dip coating and deposition techniques^41,42^. The development of PSHE measurement techniques^22–25^, opens the possibilities of designing unconventional Hardware security primitives in the future.

## Conclusion

In conclusion, our study presents a novel method utilizing the Photonic Spin Hall Effect (PSHE) within a 1D Photonic Crystal (1D-PhC) to develop Physically Unclonable Functions (PUFs). These PUFs leverage the PSHE effect to generate unique Challenge–Response pairs (CRPs). By employing PSHE effects through analytical simulations on a 1D-PhC, we expand the design possibilities for PUFs. Our proposed PUFs demonstrate promising performance metrics, including an Inter-Hamming distance of 47.50%, an average proportion of unique responses of 62.5%, and a Pearson correlation coefficient of − 0.198. Moreover, the PUF token exhibits robustness to simulated noise. Security assessment using a Machine Learning (ML)-based attack model reveals a low average accuracy of successful attack prediction (9.70%), indicating resilience against MLP-based attacks. However, further optimization is needed for parameters such as Inter-Hamming distance and bit distribution to enhance PUF structures. While a silicon proof of concept was not included, future work will involve fabricating our PUF designs on silicon chips and conducting extensive reliability (using intra-hamming distance), uniqueness, uniformity, randomness (using NIST statistical suits), and security tests (side-channel analysis). This will include environmental variation tests and resistance evaluations against attacks. These steps will validate our designs on actual hardware and demonstrate their practical applicability in real-world scenarios. Overall, our study highlights the potential of PSHE-based PUFs in hardware security, emphasizing their non-linearity, resilience, sensitivity to process variation, and easier fabrication and characterization using existing methods.

## Data Availability

The datasets used or analyzed during the current study are available from the corresponding author (A.K.G.) upon reasonable request.
